# Use of concentrated bone marrow aspirate and platelet rich plasma during minimally invasive decompression of the femoral head in the treatment of osteonecrosis

**DOI:** 10.3325/cmj.2013.54.219

**Published:** 2013-06

**Authors:** John R. Martin, Matthew T. Houdek, Rafael J. Sierra

**Affiliations:** Department of Orthopedic Surgery, Mayo Clinic, Rochester, MN, USA

## Abstract

The aim of this paper is to describe our surgical procedure for the treatment of osteonecrosis of the femoral head using a minimally invasive technique. We have limited the use of this procedure for patients with pre-collapse osteonecrosis of the femoral head (Ficat Stage I or II). To treat osteonecrosis of the femoral head at our institution we currently use a combination of outpatient, minimally invasive iliac crest bone marrow aspirations and blood draw combined with decompressions of the femoral head. Following the decompression of the femoral head, adult mesenchymal stem cells obtained from the iliac crest and platelet rich plasma are injected into the area of osteonecrosis. Patients are then discharged from the hospital using crutches to assist with ambulation. This novel technique was utilized on 77 hips. Sixteen hips (21%) progressed to further stages of osteonecrosis, ultimately requiring total hip replacement. Significant pain relief was reported in 86% of patients (n = 60), while the rest of patients reported little or no pain relief. There were no significant complications in any patient. We found that the use of a minimally invasive decompression augmented with concentrated bone marrow and platelet rich plasma resulted in significant pain relief and halted the progression of disease in a majority of patients.

Osteonecrosis of the femoral head (ONFH) occurs when the cells of the trabecular bone and marrow in the femoral head spontaneously die, leading to fracture and collapse of the articular surface (1,2). In the US, every year ONFH occurs in 10 000-20 000 adults between the ages of 20 and 60 (1,3,4). Once collapse occurs, severe pain ensues, and the disease course rarely regresses (5-8). In order to halt disease progression and provide pain relief, 80% of patients suffering from ONFH will require a total hip arthroplasty (THA); typically at a younger age than patients undergoing a THA for osteoarthritis (9-11).

Although ONFH is a common indication for THA, the etiology of the disease is still unknown (12,13). ONFH is thought to be a multifactorial disease, with patients reporting a history of exposure to one or more risk factors, including trauma to the hip, alcohol abuse, corticosteroid use, hemoglobinopathies, pregnancy, coagulopathies, organ transplant, chemotherapy, Caisson disease, HIV, and autoimmune conditions; however in some patients the risk factor remains unknown, and the disease is termed “idiopathic” ONFH (12-16). Recent studies looking at the gentics risks of ONFH have resulted in identifying an autosomal dominant mutation in collagen type II gene (COL2 A1 gene) (17); which has been associated with genetic polymorphisms in alcohol metabolizing enzymes and the drug transport proteins (18,19).

If the disease course is recognized before collapse of the subchondral bone and cartilage, patients can be treated with core decompression of the femoral head including Ficat Stage I or II (12,20,21). This technique has been used for over four decades, however randomized control trials have failed to show that this procedure alone halts disease progression and collapse (4). Recently, concentrated bone marrow autograft has been used to augment the decompression site to attempt to repopulate the femoral head with human mesenchymal stem cells (hMSC) (13,22,23). This aim of this paper is to describe our surgical technique and early clinical results using autologous bone marrow concentrate with platelet rich plasma and a minimally invasive decompression for the treatment of ONFH.

## Surgical technique

**Figure 1:**
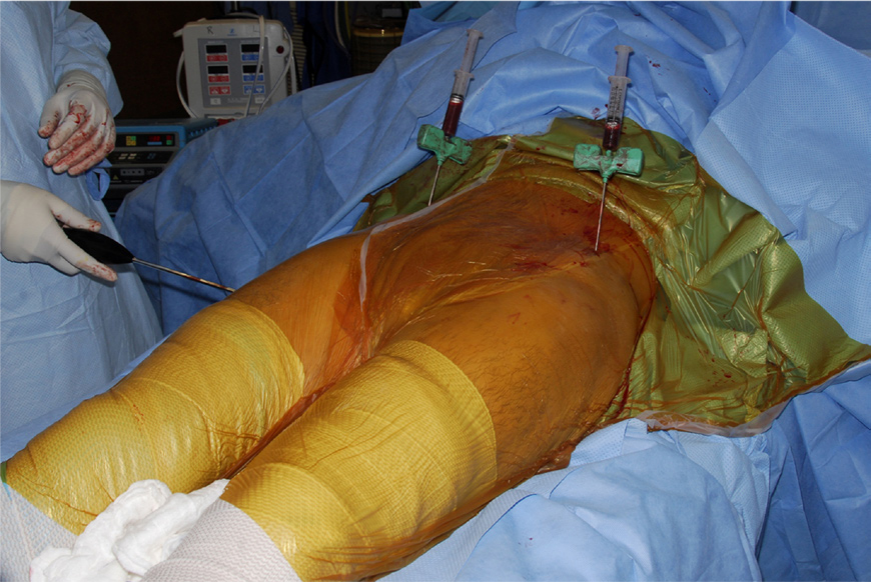
Patient with iliac crest aspirators in place, with bilateral hips prepared for decompression and injection of concentrated bone marrow (published with permission from Sierra RJ). Alternative procedure for osteonecrosis of the femoral head, Figure 2 (24).

The patient is placed supine on a radiolucent table and general anesthesia is performed. Fluoroscopic imaging is used at this time to ensure that adequate anterior-posterior (AP) and frog-leg lateral images can be obtained with the patient’s position. The operative hip/hips and both iliac crests are prepped and draped into the surgical field in a sterile manner (Figure 1). Two grams of cefazolin should be given at this time. The procedure begins by obtaining iliac bone marrow aspirate. A 2-3-mm incision is made over that anterior aspect of each anterior iliac crest. When the iliac crest is visible, the trochar (Marrowstim, Biomet Biologics, Warsaw, IN, USA) can be inserted into the iliac crest in between the two tables of ilium and bone marrow can be aspirated into 10 mL-syringes. We use the Bio-Cue System (Biomet Biologics) to concentrate the bone marrow aspirate obtained. Each vial requires 57 mL of bone marrow aspirate mixed with 3 mL of heparin to prevent clot formation. A single vial produces 6 mL of concentrated bone marrow aspirate, and we typically utilize two vials per hip. Once collected, the vials are placed in a centrifuge for 15 minutes to adequately concentrate the mononuclear fraction of blood that contains the mesenchymal progenitor cells. Additionally, 120 mL of anticoagulated blood are placed into two additional separate vials and centrifuged for 15 minutes. Approximately 12 mL of platelet rich plasma is obtained.

## Hip decompression

**Figure 2:**
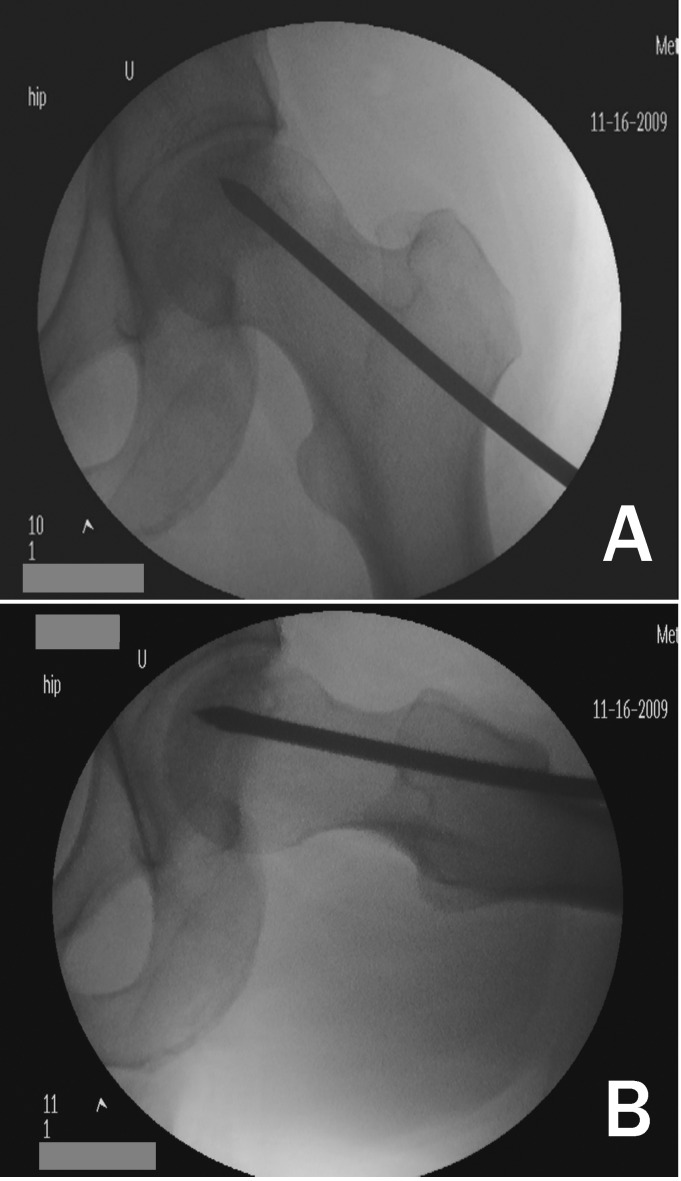
Anteroposterior (**A**) and frog-leg lateral (**B**) radiograph showing the decompression trochar in the area of necrosis (published with permission from Sierra RJ). Alternative procedure for osteonecrosis of the femoral head, Figures 3A and 3B (24).

While the bone marrow and blood are being centrifuged, we shift our attention to the hip decompression. A 1-cm incision is made over the lateral aspect of the femur just below the vastus ridge of the trochanter. The position should be confirmed by fluoroscopy. Typically power instruments are not necessary except if entry into the lateral cortex is difficult. The starting point is maintained proximal to the level of the lesser trochanter and distal to the vastus ridge. When the ideal starting point has been obtained the trochar is advanced from lateral to medial under biplanar fluoroscopy; taking care to verify the position of the trochar as the tip needs to end “in” the necrotic lesion. A 6-mm trochar is utilized for the decompression. The trochar is advanced into the necrotic lesions with gentle mallet taps. Typically, a change in pitch is noted when the trochar has reached the area of necrosis (Figure 2). The areas of necrosis can be entered with the trochar but should not be advanced within 5 mm of subchondral bone to avoid collapse. Correct trochar positioning is confirmed under biplanar fluoroscopy. The inner core of the trochar is removed, leaving the 6 mm trochar in the necrotic portion of the femoral head.

## Injection of concentrated bone marrow

**Figure 3:**
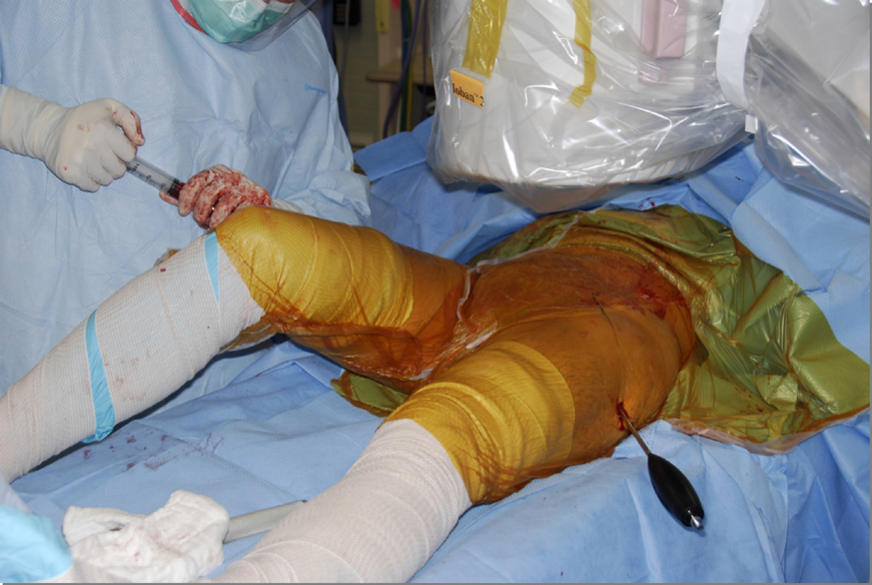
Injection of bone marrow concentrate into the area of necrosis (published with permission from Sierra RJ). Alternative procedure for osteonecrosis of the femoral head, Figure 4 (24).

At this time, the contents of the bone marrow concentrate and the platelet-rich plasma have completed the centrifugation process. Approximately 12 mL of each should be obtained. They are combined in a 30 mL-syringe in a sterile manner. This can be doubled if both hips are to be injected. The contents of the 30 mL-syringe are then injected into the trochar, which should be positioned in the necrotic lesion of the femoral head. Due to the sclerotic nature of the lesions, it may require significant pressure to inject the contents from the syringe (Figure 3). If excessive resistance is met, the trochar can be retracted while confirming that the tip remains in the area of necrosis. This will increase the space available to inject while ensuring that the mesenchymal stems cells are delivered to the correct area. After injecting the contents of the syringe, the bone marrow/PRP solution will likely flow retrograde due to pressure gradients. To prevent this retrograde backflow, the trochar can be removed, and reinserted into the previous track at a different angle in order to push cancellous bone into the tract.

## Post-operative care

All patients were discharged home the day of surgery. Patients are allowed to weight bear as tolerated with the use of crutches for approximately 2 weeks. In patients that undergo bilateral procedures, we recommend the use of crutches until hip pain subsides, allowing for full pain-free ambulation. Some patients have an increase in pain initially after the injection. This pain is unlikely to last more than 3 months and the majority of patients have significant pain relief within a few weeks. Because the technique described utilizes a smaller trochar than is typically used for core decompression, it allows patients to weight bear as tolerated immediately postoperatively. Patients are discharged on 10 mg of Simvastatin daily until healing or collapse of the lesion occurs. We found that many patients only use the crutch for the first week.

## Our results

**Figure 4:**
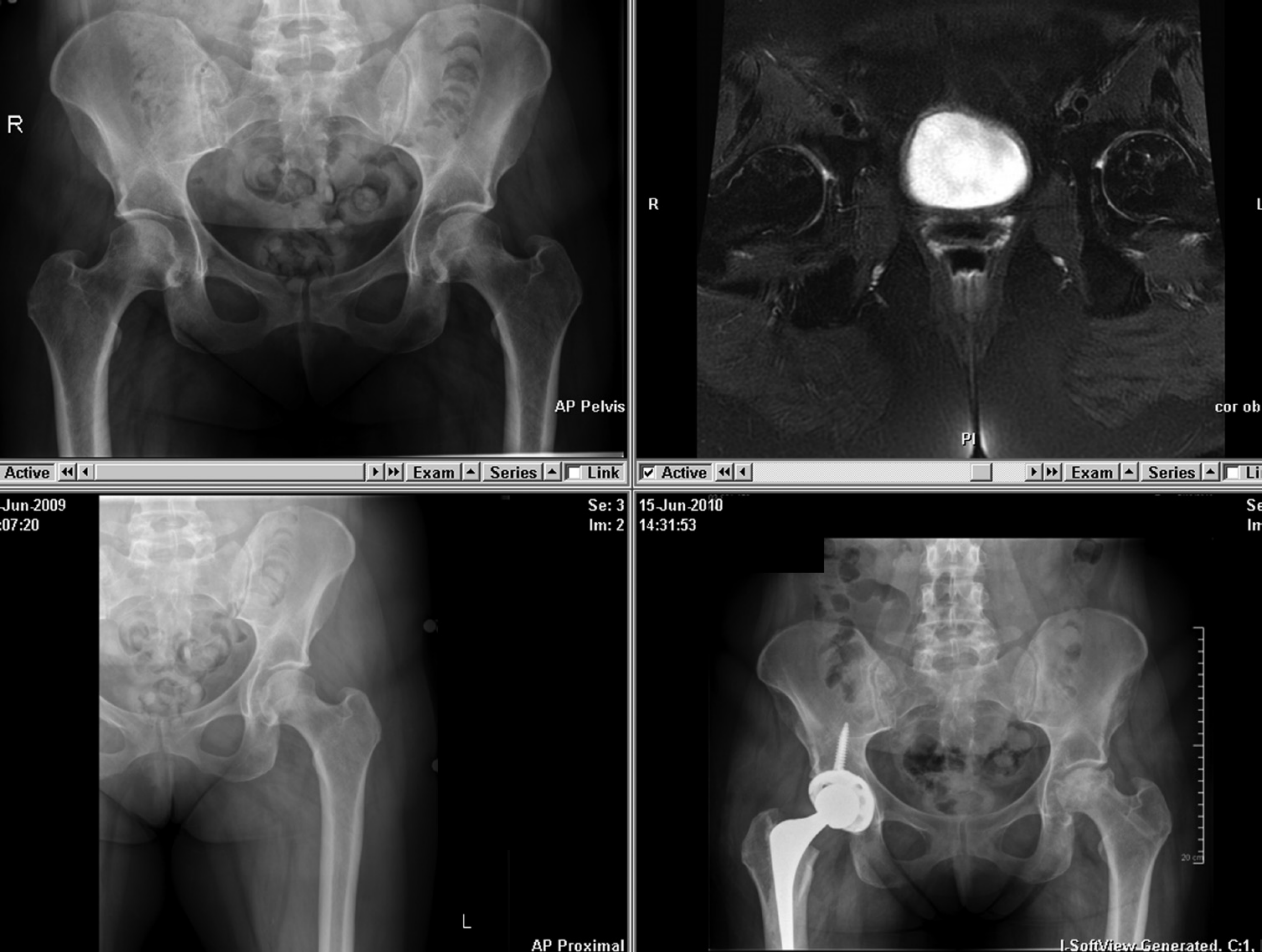
A 43-year-old female on steroids for cancer treatment presents with bilateral hip involvement. Upper left: anteroposterior (AP) pelvis radiograph taken on presentation. Lower left: AP of the left femur taken on presentation. Upper right: T2 coronal oblique MRI showing significant bilateral head involvement prior to treatment. Lower right: AP pelvis shows complete collapse of the left femoral head resulting in total hip arthroplasty 1 year post-operative.

At our institution, hip decompression with the introduction of concentrated bone marrow and platelet rich plasma was performed on 73 hips. We retrospectively reviewed this patient population under institutional review board (IRB) approval from our institution. Patients consented to current research study under the protocol set forth by the IRB committee. Patients were followed for an average of 17 months. Twenty-five patients underwent unilateral decompression and 24 underwent bilateral decompression. Preoperative x-rays and MRI was obtained on all patients. Thirty-six left hips and 37 right hips underwent decompression. The average age of patients was 43 years. Fifty-seven patients had Stage I and 16 patients had stage II. Four hips were re-injected due to continued symptoms without radiographic evidence of progression. Sixteen hips (21%) progressed to further stages of osteonecrosis, ultimately requiring total hip replacement (Figure 4). There were no significant complications in any patient undergoing this procedure. Two patients were noted to have post-operative trochanteric bursitis at immediate follow-up, however this was managed non-operatively. The top three underlying etiologies included steroids (n = 44), alcohol (n = 10), and idiopathic ONFH (n = 9). Significant pain relief was reported in 86% of patients (n = 60), while the rest of patients reported little or no pain relief.

## Original description

The introduction of mesenchymal stem cells into the femoral head for avascular necrosis was originally described in 2002 (22). At this time, physicians were disappointed with the results of a standard core decompression and believed that the introduction of bone marrow concentrate could introduce mesenchymal stem cells into an area of necrosis. A retrospective review of 116 patients and 189 hips described a technique that utilized a trephine approach to enter the area of necrosis under fluoroscopy and then inject concentrated bone marrow directly into this area. It found excellent results in patients who were pre-collapse (stage I or II). In this group, 9 out of 145 hips required a total hip arthroplasty at minimum five-year follow-up (22). However, in patients who had already collapsed (stage III or IV), 25 out of 44 hips required a THA. The study also evaluated the underlying etiology that predisposed the patient to AVN by correlating the etiology of necrosis to the need for subsequent THA and the number of mesenchymal stem cell colony forming units (22). The three most common factors were sickle cell disease, alcohol use, and corticosteroid use (22).

## Randomized trials

The original work by Hernigou was a retrospective study that popularized the addition of bone marrow concentrate to a core decompression (22). The information obtained from the study have been proven very beneficial in identifying the procedure and etiologic data, however the study did not compare the results with core decompression in a prospective manner. Since this study, at least 4 studies have prospectively compared the results of standard core decompression and core decompression with autologous bone marrow introduction.

In 2004, Gangji et al in a prospective randomized controlled trial compared the results of core decompression with core decompression with bone marrow (CDBM) (23). The study specifically looked at patients with stage I-II AVN, and excluded all patients with post-collapse AVN. Eight hips underwent core decompression and 10 hips underwent CDBM. The patients’ age and underlying cause of AVN were similar. During the 24-month period, the CDBM group had a statistically significant decrease in pain (*P* = 0.021). The Lequesne and WOMAC indices were also significantly improved. At follow-up, 5 of 8 hips in the core decompression group collapsed compared to only 1 of 10 in the CDBM group. The author also found that the volume of involvement of AVN of the femoral head in the CDBM group had significantly decreased from 15.6% pre-op to 10.1% at 24 months. In the core decompression group, it significantly increased from 16.7% pre-op to 20.6% (*P* = 0.036). Finally, both methods were found to have no major complications (23). This paper was followed-up in 2011 with 5-year of clinical follow-up (25). At the 5-year time point, 8 of 11 hips in the core decompression group progressed to fracture and collapse, while in the CDBM group only 3 of 13 progressed to collapse (25).

Chang et al conducted a prospective trial, which included 14 hips that underwent core decompression or CDBM (26). The randomization included involvement of the left or right hip. CDBM group had a significantly lower Harris Hip score and area of femoral head necrosis (*P* < 0.05). One patient in the core decompression group collapsed compared to no patients in the CDBM group. No significant complications were noted in either group. Due to the small number of patients in each group, it is difficult to draw any definitive conclusions from this study.

In a recent prospective trial by Sen et al, 25 hips underwent core decompression and 26 hips underwent CDBM (27). All patients were followed for a minimum of two years. At the final follow-up, the CDBM group had significantly higher Harris Hip Scores. They found no difference in radiographic or MRI improvement between the groups. The authors also noted that pre-operative etiologies also significantly affected outcome and that patients with poor pre-op Harris Hip score, x-ray changes, edema, and effusion on MRI had better results in the CDBM group.

Zhao et al also looked at a similar group of patients (13). Fifty-one hips underwent core decompression and 53 hips underwent CDBM. Ten patients in the core decompression group progressed and eventually required THA or vascularized fibula graft. Only two hips in the CDBM group required vascularized fibula graft. Patients who underwent CDBM also had a significantly higher Harris Hip score at the final follow-up. All groups excluding patients with pre-operative IIa lesions in the core decompression group had a significant decrease in volumetric involvement of the femoral head. No significant complications were found in either group. They also concluded that the size and the location of the AVN lesion was likely the most important factor in patient outcomes.

In conclusion, the combination of hip decompression and injection of mesenchymal stem cells into the necrotic lesion provides satisfactory results in patients with early stage ONFH and can lead to complete resolution of the necrotic lesion. The procedure is simple, with a low complication rate and the patients are allowed to weight bear as tolerated allowing them an early return to function and activities of daily living.
